# Autistic traits in trichotillomania

**DOI:** 10.1002/brb3.2663

**Published:** 2022-06-08

**Authors:** Jon E. Grant, Samuel R. Chamberlain

**Affiliations:** ^1^ Department of Psychiatry & Behavioral Neuroscience University of Chicago Pritzker School of Medicine Chicago Illinois USA; ^2^ Department of Psychiatry Faculty of Medicine University of Southampton Southampton UK; ^3^ Southern Health NHS Foundation Trust Southampton UK

**Keywords:** autism, impulsivity, obsessive‐compulsive disorders

## Abstract

**Introduction:**

Although many variables have been examined as potentially contributing to the manifestation of trichotillomania (TTM), little research has focused on problems in social interactions. Hair pulling has many similarities to the stereotypies seen in autism spectrum disorders (ASD), and thus the present study examined autistic traits in adults with trichotillomania.

**Methods:**

Fifty nontreatment‐seeking adults with DSM‐5 TTM were recruited. Participants completed standard diagnostic interviews, basic demographic information, and symptom inventories about TTM. Autistic traits were quantified using the Brief Autism‐ Spectrum Quotient (AQ‐10) which screens for autistic traits.

**Results:**

The sample comprised 50 participants, mean (standard deviation) age of 30.2 (5.6) years, 10% being male, 86% female, and 4% nonbinary. Eight of the participants had a history of major depressive disorder and six had a history of an anxiety disorder. No one had current or lifetime obsessive‐compulsive disorder. The mean AQ10 score was 3.5 (2.0), with 14.6% scoring 6 or greater. Autism scores correlated significantly only with family dysfunction and not with symptom severity or impulsivity.

**Conclusions:**

This study examined autistic traits in a community‐based sample of adults with TTM and found elevated rates of probable ASD (based on a self‐report screening tool) among those with TTM. These results highlight the need to carefully screen for autistic traits in those with TTM. To what extent these traits may influence response to treatment, however, remains unclear.

## Abstract 2

This study found that autistic traits were common in adults with trichotillomania and associated with family dysfunction. These results highlight the need to carefully screen for autistic traits in those with TTM. To what extent these traits may influence response to treatment, however, remains unclear.

1

Although many variables have been examined as potentially contributing to the manifestation of trichotillomania (TTM), little research has focused on problems in social interactions. Hair pulling has many similarities to the stereotypies seen in autism spectrum disorders (ASD), and is not uncommon in those with ASD. In fact, a study of 112 children with ASD found that the 3‐month point prevalence of TTM was 3.9% (Simonoff et al., 2008), a rate almost twice that found in the general population (Grant et al., 2020). Examination of traits representing stereotypy and deficits in social interactions may be beneficial to understand vulnerability markers and the relationship between social deficits and habit behaviors such as TTM. Thus, the present study examined autistic traits in adults with TTM.

Fifty nontreatment‐seeking adults with DSM‐5 TTM were recruited via advertisements for a study on TTM. The advertisement text did not mention autism or autistic traits. Inclusion criteria were age 18–65 years and meeting DSM‐5 criteria for TTM. Subjects were excluded if they were unable to give informed consent or were unable understand/undertake the study procedures. All study procedures were carried out in accordance with the Declaration of Helsinki. The Institutional Review Board of the University of Chicago approved the study and the consent statement. Participants were compensated with a $50 gift card for a local department store.

Participants completed standard diagnostic interviews, basic demographic information, and symptom inventories about TTM. Autistic traits were quantified using the Brief Autism‐Spectrum Quotient (AQ‐10) which screens for self‐reported autistic traits (Baron‐Cohen et al., 2021). A cut‐off score of 6 or more out of 10 is used to identify people who have a likely diagnosis of autism. Other measures comprised: the self‐report Massachusetts General Hospital Hair Pulling Scale (MGH‐HPS), Sheehan Disability Scale (SDS), 24‐item Hamilton Depression Rating Scale (HAM‐D), Hamilton Anxiety Rating Scale (HAM‐A), and the Barratt Impulsivity Scale‐11 (BIS‐11).

Relationships between AQ‐10 scores and the other measures of interest were characterized using Pearson's correlations. All analyses were conducted using JMP Pro software and significance was defined as *p* < .05 uncorrected.

The sample comprised 50 participants, mean (standard deviation) age of 30.2 (5.6) years, 10% being male, 86% female, and 4% nonbinary. Eight of the participants had a history of major depressive disorder and six had a history of an anxiety disorder. No one had current or lifetime obsessive‐compulsive disorder. The mean level of education was indicative of at least some college education. The mean AQ10 score was 3.5 (2.0), with 14.6% scoring 6 or greater. AQ10 scores did not differ significantly between men and women (*F* = .148, *p* = .863) or as a function of ethnicity (*F* = .424, *p* = .861). An overview of correlations between AQ10 scores and the other measures is displayed in Table 1.

**TABLE 1:** Relationship of autistic traits to demographic and clinical measures

Against AQ total score	Pearson's correlations	
	Correlation	*p*
Age, years	−.0941	.5245
Education level	.0956	.5181
MGH‐HPS	.1881	.2004
SDS‐Work	.2177	.1371
SDS‐Social life	.0634	.6686
SDS‐Family	.3496	.0149
HAM‐A	.1014	.4929
HAM‐D	−.0345	.8161
BIS attentional impulsivity	.1286	.3837
BIS motor impulsivity	−.1252	.3965
BIS nonplanning impulsivity	.0244	.869

Abbreviations: AQ, autism quotient; BIS, Barratt Impulsivity Scale; HAM‐A, Hamilton Anxiety Rating Scale; HAM‐D, Hamilton Depression Rating Scale; MGH‐HPS, Massachusetts General Hospital Hair Pulling Scale; SDS, Sheehan Disability Scale.

* significant at *p* < .05.

This study examined self‐reported autistic traits in a community‐based sample of adults with TTM and found that 14.6% of the sample scored in the range suggestive of autism. Caution is needed when comparing this rate to external norms because normative samples may differ from the current clinical sample in a number of important ways, and studies differ in methodological approaches too. However, consideration of normative data may be informative. In a large general population sample (from Stockholm), 6–10% of participants met the AQ‐10 threshold for probable autism (Lundin et al., 2019). In large population datasets, prevalence of ASD in the USA was estimated to be 2–2.5% (Dietz et al., 2020), and in the UK, around 1% (Brugha et al., 2011). As such, the rate seen here in TTM appears higher than might be expected. This suggests that follow‐up work should investigate the potential link between TTM and ASD using a larger study and rigorous diagnostic interviews. It should be noted that we refer to these as traits since the study did not use clinical interviews, which would be needed to diagnose ASD formally. Autism scores correlated significantly only with family dysfunction and not with symptom severity or impulsivity.

Several limitations should be considered. Because this study was conducted in community‐recruited participants with TTM, the findings may not generalize to clinical populations. Because the study was cross‐sectional, direction of effect cannot be shown, although of course autistic tendencies could be present prior to TTM (i.e., autistic traits may increase propensity for developing TTM). Although categorical obsessive‐compulsive disorder was not present in the participants, we failed to assess for obsessive‐compulsive symptoms as well as sensory sensitivities, both of might be influencing these findings as they are relatively common among patients with TTM and also share some overlap with ASD features. Although a sample size of 50 may be sufficient to detect correlations, follow‐up work in larger samples would be valuable. The current study did not correct for multiple comparisons, as this would have resulted in unacceptably low statistical power to detect true positive effects with this sample size. Future larger sample sizes would enable correction for multiple comparisons while retaining adequate statistical power. We focused on self‐reported traits (defined using a questionnaire) rather than formal diagnoses. Additionally, the AQ‐10 tool has psychometric limitations and has been critiqued for lack of coverage of restrictive‐repetitive behaviors (Taylor et al., 2020).

In conclusion, this study found that 14.6% of adults with TTM met threshold on a self‐report questionnaire suggestive of ASD; and that scores on questionnaire were significantly associated with extent of family dysfunction (with medium effect size). These results highlight the need to carefully screen for autistic traits in those with TTM. To what extent these traits may influence response to treatment, however, remains unclear. Future work should measure whether autistic traits in TTM might be accounted for by comorbidities or particular phenomena (e.g., obsessive‐compulsive symptoms or sensory sensitivities related to the hair pulling). It should also extend these initial findings to larger samples using rigorous diagnostic tools.

## CONFLICT OF INTEREST

This study was funded by internal funds. Grant has received research grants from Otsuka and Biohaven Pharmaceuticals. He receives yearly compensation from Springer Publishing for acting as Editor‐in‐Chief of the Journal of Gambling Studies and has received royalties from Oxford University Press, American Psychiatric Publishing, Inc., Norton Press, and McGraw Hill. Chamberlain's involvement in this research was funded by a Wellcome Trust Clinical Fellowship (110049/Z/15/Z). He receives a stipend from Elsevier for editorial work.

### PEER REVIEW

The peer review history for this article is available at https://publons.com/publon/10.1002/brb3.2663

## Data Availability

The data that support the findings of this study are available from the corresponding author upon reasonable request and the creation of a data sharing agreement.
